# Molecular interaction mechanisms of muscle fiber type transition in aging sarcopenia and the role of exercise intervention

**DOI:** 10.1186/s43162-026-00698-9

**Published:** 2026-08-27

**Authors:** ShaoHui Zhang, Jialin Zhang, Tao Fu, Linliang Zhao, Kexin Li, Sifan Yan

**Affiliations:** 1 Tianjin Key Laboratory of Exercise Physiology and Sports Medicine, Institute of Sport, Exercise ＆ Health, Tianjin University of Sport, Tianjin, China; 2 Tianjin Sport University College of Competitive Sports, Tianjin, China

**Keywords:** Sarcopenia, Muscle fiber type transition, Molecular mechanisms

## Abstract

Muscle fiber type transition is a core pathophysiological process in aging sarcopenia; however, existing research has often analyzed its underlying mechanisms in isolation. This review systematically characterizes the features of muscle fiber type transition in sarcopenia at the neural, cellular, and microenvironmental levels, and dissects the multidimensional molecular interaction mechanisms, including disrupted neural input, aberrant intracellular protein metabolism, and inflammaging. Based on this mechanistic framework, we propose exercise intervention strategies targeting these molecular pathways, emphasizing the core principles of stage-specific adaptation to the aging process and phenotypic matching of fiber damage characteristics. Furthermore, we elaborate on the molecular mechanisms underlying resistance training, aerobic exercise, and high-intensity interval training. This review offers a novel perspective for the personalized prevention and treatment of sarcopenia and provides a theoretical foundation for the future development of biomarkers and the formulation of precise exercise prescriptions.

## Introduction

Age-related sarcopenia refers specifically to the physiological process associated with aging, characterized by a reduction in the cross-sectional area of muscle fibers, a transition in muscle fiber types, and a decline in both muscle strength and endurance. This concept was first proposed by Rosenberg [55]. In 2016, sarcopenia was included in the International Classification of Diseases with its own unique code (ICD-11) [2]. Subsequently, several major research consortia were established worldwide, including the Asian Working Group for Sarcopenia (AWGS) [8], the European Working Group on Sarcopenia in Older People (EWGSOP) [11], and the International Working Group on Sarcopenia (IWGS) [18]. Notably, the Global Leadership Initiative in Sarcopenia (GLIS) has updated its core definition, characterizing sarcopenia as an age-related, systemic skeletal muscle disorder. Its core features include reduced muscle mass, decreased muscle strength, and diminished muscle power, whereas impaired physical performance and limitations in activities of daily living are considered common outcomes rather than defining components [34]. The 2025 consensus from the Asian Working Group for Sarcopenia (AWGS) further extends the diagnostic scope to include middle-aged adults aged 50–64 years, simplifies the diagnostic criteria to the simultaneous presence of low muscle mass and low muscle strength, and designates physical performance as an outcome measure, thereby providing a robust rationale for early intervention [7].

The pathogenesis of sarcopenia is multifactorial, primarily involving imbalances in protein synthesis and degradation [21], oxidative stress [59], inflammatory responses [41], micronutrient homeostasis [38], and cellular senescence [79]. Izquierdo et al. reported that after the age of 60, muscle mass declines at a rate of 1%–2% per year in older adults, with the rate accelerating to 3%–5% after the age of 70 [29]. Muscle power also exhibits an accelerated decline with advancing age, decreasing by 40%–50% in individuals aged 50–70 years, and by over 60% after the age of 70 [5, 6]. Sarcopenia is characterized not only by reduced muscle mass and functional decline but also by alterations in muscle fiber type composition [49]. Skeletal muscle fibers are classified into type I, type IIa, type IIx, and hybrid fibers based on myosin heavy chain (MyHC) isoforms. This physiological and functional heterogeneity determines the plasticity of muscle fibers throughout their lifespan. Under normal physiological conditions, muscle fiber transition follows a bidirectional gradient pattern: type I ↔ type IIa ↔ hybrid ↔ type IIx [67]. Specifically, endurance training induces a type IIa → type I transition, whereas resistance training induces a type I → type IIa transition [39]. During the natural aging process, the predominant transition pattern is the conversion of type II fast-twitch fibers toward type I slow-twitch fibers, with the type IIx → type IIa → type I gradient being particularly pronounced [36]. This phenomenon, characterized by the preferential loss of fast-twitch fibers and the pathological transition of slow-twitch fibers, represents one of the key pathological drivers of the decline in muscle mass and the heterogeneity of muscle function.

Existing research has predominantly employed isolated and static analyses of individual mechanisms underlying muscle fiber type transition, lacking a systematic dissection of the interplay among neural input, cell-autonomous regulation, and the muscular microenvironment. Furthermore, investigations into the regulation of irreversibility and spatial heterogeneity of this transition remain insufficient. This review systematically discusses the characteristic features of muscle fiber type transition in sarcopenia, provides an in-depth analysis of its underlying mechanisms, and proposes exercise intervention strategies, thereby offering a novel perspective for the prevention and treatment of sarcopenia.

## Literature search strategy and methods

### Literature search strategy

A computerized literature search was conducted in the English databases PubMed, Web of Science, and Embase, as well as the Chinese database China National Knowledge Infrastructure (CNKI). The search period was limited to January 1995 to May 2026. Search terms included sarcopenia, muscle fiber type transition, exercise intervention, and molecular mechanisms. The search was restricted to human clinical studies and rodent basic research. Eligible study types included randomized controlled trials, cohort studies, and basic mechanistic studies. Case reports and duplicate publications were excluded.

### Literature screening and quality assessment

Inclusion criteria were as follows: (1) explicit elucidation of muscle fiber type transition in sarcopenia; (2) involvement of molecular mechanisms at the neural, cellular, or microenvironmental levels; and (3) inclusion of exercise intervention effects. Exclusion criteria were: (1) pure commentary without original data; (2) ambiguous mechanistic analysis; and (3) clinical studies with a sample size of less than 5.

A total of 81 articles were ultimately included, of which 43 were human studies and 38 were mouse/rat studies. Findings from animal studies in this review are labeled as basic evidence only, and extrapolation to humans requires cautious validation.

### Data extraction

Core data, including study subjects, experimental models, molecular pathways, and changes in muscle fiber subtypes, were independently extracted by two researchers to ensure consistency in data extraction.

## Molecular interaction mechanisms driving muscle fiber type transition

### Neural input disruption and neuromuscular junction degeneration

The integrity of neuromuscular communication is a fundamental determinant of muscle fiber type specificity. In sarcopenia, disrupted neural input initiates the denervation and transition program in type IIx fibers by selectively impairing the neural pathways that innervate fast-twitch fibers. Furthermore, this process acts synergistically with satellite cell dysfunction to create an amplification effect [61].

#### Preferential loss of large-diameter motor neurons

Large-diameter α-motor neurons (MNs) that innervate type IIx fibers are characterized by highly complex axonal terminal arborizations. Each of these neurons innervates over 1,000 muscle fibers and projects over long distances to reach distal limb muscles, resulting in significantly higher maintenance demands compared to the small-diameter MNs that innervate type I fibers. Specifically, type IIx muscle fibers are primarily glycolytic and rely on large, infrequent neural impulses, a pattern that is more prone to oxidative stress than the continuous low-amplitude activity of type I fibers. Type I fibers, with their high mitochondrial density and oxidative capacity, possess greater energy buffering and antioxidant defense capabilities, rendering them more resilient under various stress conditions. However, large-diameter MNs lack comparably robust oxidative stress defense mechanisms, making them preferentially vulnerable to degeneration during aging. Owing to their elevated metabolic requirements and susceptibility to oxidative stress, these neurons become preferential targets of degeneration in the aging process [61].

From a molecular perspective, the stability of the microtubule cytoskeleton in long axons is dependent on the transport system mediated by dynein and kinesin. During aging, the expression levels of these motor proteins are significantly downregulated, accompanied by aberrant phosphorylation modifications. This directly leads to impaired retrograde transport of neurotrophic factors and uneven mitochondrial distribution at axon terminals, ultimately resulting in energy depletion and structural disintegration of the axon terminals. Furthermore, the physiological recruitment pattern of type IIx fibers exacerbates the vulnerability of large-diameter MNs. Type IIx fibers are selectively recruited only during explosive movements and are activated infrequently during routine daily activities. Consequently, the innervating MNs fail to receive sufficient neural activity-dependent survival signals, further compromising their anti-apoptotic capacity. Concurrently, reactive oxygen species (ROS) attack DNA, generating oxidative damage products such as 8-hydroxy-2'-deoxyguanosine (8-OHdG), the accumulation of which leads to genomic instability [1, 61].

The myelin sheaths of large-diameter MNs are prone to loss and degradation. Degradation products, such as myelin basic protein fragments, activate microglia to secrete pro-inflammatory cytokines including IFN-γ and TNF-α. These cytokines bind to corresponding receptors on the surface of MNs and, via activation of the NF-κB signaling pathway, trigger an inflammatory cascade within the neurons, accelerating axonal dystrophy and somatic apoptosis [16, 35]. Lai et al., using single-cell transcriptomic data, further characterized the molecular features associated with this pathological process. In aging skeletal muscle, the expression of MN-associated genes encoding fast-twitch fiber-specific neurotransmitter release proteins, such as SNAP25 and Syntaxin 1 A, was significantly downregulated in MNs innervating type IIx fibers. Moreover, the extent of MN loss was significantly positively correlated with the degree of type IIx myonuclear loss [36].

**Fig. 1:**
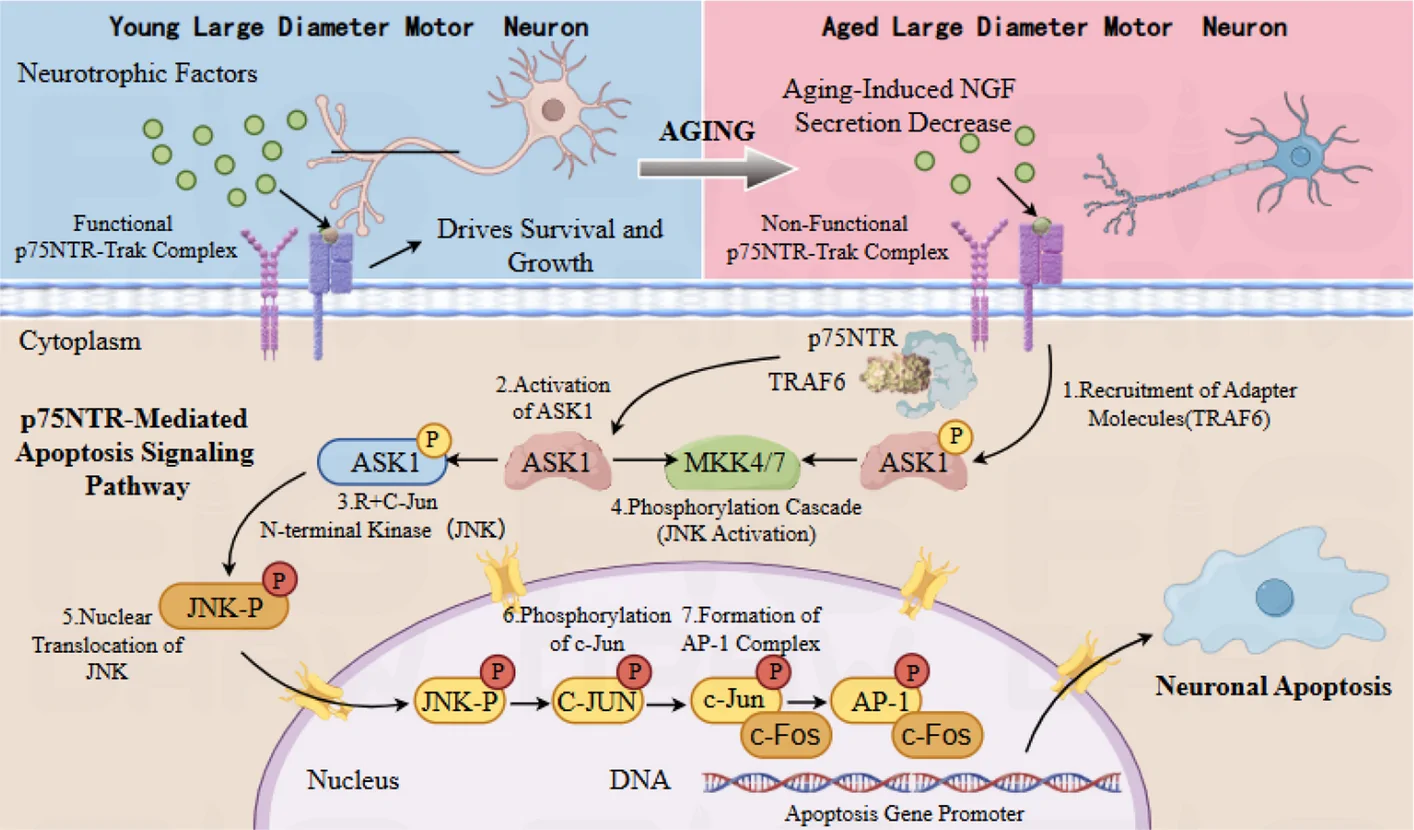
Mechanism of neuronal apoptosis induced by aging

The FOXO transcription factor family, particularly FOXO3a, plays a critical regulatory role in MN survival. During aging, FOXO3a undergoes phosphorylation-mediated inactivation, which prevents its nuclear translocation and subsequent activation of anti-apoptotic gene expression, while also reducing its transcriptional regulation of genes involved in oxidative stress defense [28]. Conversely, the mTOR signaling pathway is hyperactivated in aging large-diameter MNs, excessively promoting protein synthesis and cell proliferation, thereby exacerbating metabolic stress and oxidative damage in these neurons [23]. Furthermore, the expression levels of neurotrophic factor receptors, including the GDNF receptor c-Ret and the BDNF receptor TrkB, are significantly downregulated in large-diameter MNs, further impairing their ability to sense and respond to muscle-derived neurotrophic signals [15]. Concurrently, age-related reductions in nerve growth factor (NGF) secretion prevent p75NTR in large-diameter motor neurons from forming functional complexes with the tyrosine kinase receptor TrkA. Instead, p75NTR recruits adaptor molecules such as TRAF6 (TNF receptor-associated factor 6), which dephosphorylates and activates apoptosis signal-regulating kinase 1 (ASK1). This is followed by phosphorylation of c-Jun N-terminal kinase (JNK). Upon nuclear translocation, phosphorylated JNK phosphorylates c-Jun, leading to the formation of the activator protein 1 (AP-1) transcription complex. AP-1 then binds to the promoters of pro-apoptotic genes, thereby inducing neuronal apoptosis [61, 68]. A detailed schematic of this process is presented in Fig. 1. Figure 1 Mechanism of Neuronal Apoptosis Induced by Aging.

#### Disintegration of neuromuscular junction structure

The neuromuscular junction (NMJ) serves as the functional hub connecting MNs and muscle fibers, and its structural integrity is critical for maintaining muscle fiber phenotype stability. During aging, the presynaptic membrane, postsynaptic membrane, terminal Schwann cells (TSCs), and synaptic cleft undergo coordinated degeneration. This not only blocks the reinnervation and repair of type IIx fibers but also consolidates their denervated state.

Specifically, the axon terminals of large-diameter MNs initially exhibit impaired vesicle release function. Aging induces a significant downregulation in the expression levels of key components of the SNARE complex, including synaptic vesicle-associated membrane proteins and SNAP25, directly compromising the quantal release efficiency of acetylcholine. This results in a reduced amplitude of endplate potentials (EPPs) and diminished synaptic transmission efficacy [76]. Concurrently, the secretion of Agrin by MNs is markedly reduced. As a key factor in maintaining NMJ stability, this deficiency of Agrin directly impairs its regulatory role in clustering acetylcholine receptors on the postsynaptic membrane [71]. The protective function of TSCs on axon terminals also deteriorates in parallel. Aging leads to a significant decline in TSC proliferative capacity and downregulation of GAP43 expression, rendering TSCs unable to effectively guide axonal sprouting for synaptic repair. Consequently, their phagocytic function in clearing degenerating axon terminals is compromised, ultimately obstructing the reinnervation process [23, 42].

On the muscle fiber side, the disintegration of the acetylcholine receptor (AChR) clustering region is closely associated with dysfunction of the MuSK-LRP4-Agrin signaling complex. Aging induces a significant decline in the phosphorylation level of LRP4 receptors on the muscle fiber surface, impairing their ability to effectively bind Agrin and activate MuSK, thereby inhibiting AChR clustering and stabilization [71]. Concurrently, the ubiquitination and degradation of AChRs are aberrantly activated. For instance, the expression level of the E3 ubiquitin ligase MuRF1 is significantly upregulated, leading to flattening of the junctional folds and a reduction in AChR density [56]. Using single-cell epigenetic data, Lai et al. further revealed that the proportion of NMJ-associated myonuclear subpopulations in type IIx fibers is significantly decreased, whereas a myonuclear subpopulation aberrantly expressing TNNT2 is enriched. This aberrant expression of TNNT2 is closely correlated with sarcomeric disorganization following muscle fiber denervation, providing further evidence for the inactivation of endplate function [36].

In addition to the reduction in Agrin, the activity of acetylcholinesterase (AChE) in the synaptic cleft is significantly enhanced during aging, which accelerates the degradation of acetylcholine and further reduces synaptic transmission efficacy [75]. Aging also induces a significant upregulation in the expression of TGF-β1 secreted by muscle fibers. TGF-β1 not only promotes fibrotic processes around muscle fibers but also exacerbates NMJ structural instability by directly inhibiting the Agrin-MuSK signaling pathway [44]. Furthermore, reduced secretion of catecholamines from autonomic nerve terminals diminishes their modulatory effect on endplate potentials (EPPs) and miniature endplate potentials (MEPPs), further compromising NMJ signal transmission [74].Although the NMJs of type I fibers also undergo age-related degeneration, their integrity is maintained for a significantly longer period compared to that of type IIx fibers. This difference may be attributed to the fact that the low-frequency but continuous electrical activity pattern of type I fibers maintains the expression levels of PGC-1α and its downstream mitochondrial biogenesis-related genes in TSCs, thereby endowing TSCs with greater metabolic adaptive capacity and resistance to oxidative stress. In contrast, the explosive, intermittent discharge pattern of type IIx fibers fails to provide equivalent homeostatic maintenance signals [36].

#### Reduction in neurotrophic support

Bidirectional neurotrophic factor (NTF) signaling between MNs and muscle fibers is fundamental to maintaining functional homeostasis of both compartments. Aging-induced impairment of this regulatory system not only accelerates the degeneration of large-diameter MNs but also promotes the phenotypic conversion of denervated type IIx fibers.

The secretion of key neurotrophic factors by type IIx fibers, including GDNF, BDNF, and CNTF, is significantly downregulated during aging [33]. Each of these factors regulates MN survival and function through specific signaling pathways. GDNF binds to the c-Ret receptor on MNs, activating the PI3K-Akt signaling pathway and suppressing the expression of the pro-apoptotic factor BAX, thereby maintaining the axonal regenerative capacity of MNs. Reduced GDNF secretion directly compromises the anti-apoptotic capacity of large-diameter MNs [64]. BDNF activates the Ras-MAPK pathway via the TrkB receptor, regulating the expression of genes involved in synaptic plasticity. Decline in BDNF levels leads to reduced functional connectivity between MNs and muscle fibers [20]. CNTF promotes MN survival and axonal extension by activating the JAK-STAT3 pathway,insufficient CNTF secretion further exacerbates MN degeneration. Using single-cell data, Lai et al. further confirmed that the expression levels of the GDNF receptor c-Ret and the BDNF receptor TrkB are synchronously downregulated in aged type IIx muscle fibers. Moreover, this change is significantly positively correlated with the reduction in the proportion of type IIx myonuclei, suggesting the presence of impaired neurotrophic signal reception [36].

The expression level of α-CGRP, a key factor secreted by large-diameter MNs that maintains endplate integrity, is significantly reduced. α-CGRP protects NMJ structural stability by inhibiting the overactivation of autophagy and the calpain system within muscle fibers; its deficiency directly compromises endplate stability [43]. In addition, axon terminals of MNs exhibit impaired retrograde transport of neurotrophic factors. Dysfunction of dynein and its auxiliary dynactin complex prevents muscle fiber-derived NTFs from being efficiently transported to the MN soma, further exacerbating the state of nutritional deprivation in MNs [69]. Furthermore, the reduction in Agrin secretion by MNs not only affects NMJ structural stability but also suppresses protein synthesis in type IIx fibers by inhibiting the activity of the mTOR signaling pathway within these fibers [23].

The reduction in neurotrophic support exacerbates oxidative stress and DNA damage in MNs through multiple pathways, resulting in the loss of more large-diameter MNs. This further loss of MNs, in turn, reduces the neurotrophic supply to muscle fibers, thereby creating a vicious cycle that triggers the phenotypic transition program in type IIx fibers. This program involves two parallel mechanisms. On one hand, activation of FOXO3a suppresses the expression of fast-twitch fiber-specific genes, such as MYH1 and ACTN3, while enhancing the transcription of slow-twitch fiber-related genes, including MYH7 and TNNT1 [46]. On the other hand, a distinct myonuclear subpopulation expressing stress-related genes, such as SAA2 and ID1, emerges. In this context, SAA2 amplifies inflammatory responses by activating the NF-κB pathway, whereas ID1 further promotes phenotypic transition by inhibiting the expression of genes involved in myogenic differentiation [36].

#### Synergistic regulatory role of satellite cells

Satellite cells (MuSCs), as the stem cell population responsible for skeletal muscle regeneration, exhibit functional abnormalities that synergistically amplify the muscle fiber transition process mediated by disrupted neural input [36, 63]. Under normal physiological conditions, satellite cells maintain quiescence by receiving neurogenic signals such as BDNF-TrkB and IGF-1-IGF-1R. However, in sarcopenia, the loss of large-diameter motor neurons leads to a deficiency of neurotrophic factors for satellite cells, causing them to prematurely enter a primed state [36]. In these primed epMuSCs, the expression of the stemness marker CALCR is downregulated, whereas the expression of immediate early genes such as FOS and JUN is upregulated. FOS and JUN form the AP-1 transcription complex, which binds to the IL-6 gene promoter and promotes the secretion of inflammatory cytokines, thereby further exacerbating the local inflammatory microenvironment [63]. Concurrently, the enrichment of TNF-α and IL-6 resulting from NMJ disintegration activates the STAT3 pathway, which promotes the ubiquitination and degradation of MyoD in satellite cells. This inhibits their differentiation into myoblasts, depriving type IIx fibers of stem cell-mediated repair and phenotype maintenance [63]. Clinical studies have confirmed that the number of satellite cells in the skeletal muscle of patients with sarcopenia is reduced by 43% compared to healthy adults, and the proportion of epMuSCs increases from 5 to 28%. This increase is significantly positively correlated with the extent of type IIx muscle fiber loss [36].

### Dysregulation of intracellular protein metabolism

Imbalance in intracellular protein synthesis and degradation homeostasis is a key molecular mechanism driving muscle fiber transition in sarcopenia [11, 55, 38]. The synergistic interplay of inhibited protein synthesis pathways, overactivated degradation pathways, and disrupted metabolic networks collectively drives the core pathological processes of sarcopenia: the selective loss of fast-twitch fibers and the transition from fast-twitch to slow-twitch fibers [52, 59].

#### Inhibition of protein synthesis pathways mediates muscle fiber type transition and functional decline

Skeletal muscle protein synthesis is fundamental to maintaining the structural integrity and phenotypic stability of muscle fibers. Age-related dysfunction of core regulatory pathways, such as mTOR and PI3K/Akt, represents a key contributing factor to muscle fiber transition in sarcopenia [80, 21].

The mTOR signaling pathway, as a central hub regulating protein synthesis, directly influences the translational efficiency of muscle fiber-specific proteins by modulating the phosphorylation of its downstream effectors: ribosomal protein S6 kinase beta-1 (S6K1) and eukaryotic translation initiation factor 4E-binding protein 1 (4E-BP1) [80]. In aging skeletal muscle, elevated oxidative stress and aberrant expression of microRNAs suppress the activity of mechanistic target of rapamycin complex 1 (mTORC1) by inhibiting the insulin-like growth factor 1 (IGF-1)/PI3K/Akt pathway [14, 57]. Studies have shown that mTORC1 activity is significantly reduced in the skeletal muscle of aged mice, leading to downregulation of fast-twitch fiber-specific myosin heavy chain IIa and IIx isoforms and a reduction in muscle fiber cross-sectional area. In contrast, the expression of the slow-twitch MyHC I isoform is relatively increased, presenting a phenotype characterized by a fast-to-slow fiber type transition [12]. Single-cell transcriptomic studies of human skeletal muscle have further confirmed that, in type II muscle fibers of nonagenarians, the expression levels of key mTOR pathway genes, such as RPS6KB1 and EIF4EBP1, are reduced by over 40% compared to young individuals, and this reduction is positively correlated with the degree of muscle fiber atrophy [36].

Aberrant activation of the transforming growth factor-β (TGF-β)/Smad signaling pathway exacerbates muscle fiber transition and fibrosis by antagonizing the myogenic program. Myostatin (MSTN), a member of the TGF-β family, inhibits the expression of myogenic regulatory factors (MRFs) by activating Smad2/3 signaling, thereby impeding the differentiation of satellite cells into fast-twitch fibers [45, 60]. In the skeletal muscle of patients with sarcopenia, TGF-β1 expression levels are elevated compared to healthy older adults, and its levels are negatively correlated with the proportion of type II muscle fibers [26]. Findings from animal model studies suggest that MSTN gene knockout significantly attenuates the loss of fast-twitch fibers in aged mice and preserves muscle fiber cross-sectional area, whereas MSTN overexpression accelerates the fast-to-slow fiber type transition and promotes muscle fibrosis [54]. However, direct causal evidence linking MSTN expression levels to muscle fiber type transition in human skeletal muscle is currently lacking,thus, the pathological significance of this signaling axis in human sarcopenia remains to be confirmed by functional studies.

Furthermore, activation of the endoplasmic reticulum (ER) stress pathway also contributes to the inhibition of protein synthesis. Age-related hypermethylation of the ER oxidoreductase 1 alpha (ERO1A) gene suppresses eIF2α dephosphorylation, thereby reducing the translational efficiency of muscle fiber-specific proteins. This effect is particularly pronounced in the synthetic metabolism of fast-twitch fibers [62, 77]. A possible explanation for this selectivity is that fast-twitch fibers rely on high translational efficiency to maintain the high expression levels of MyHC required for rapid contraction, and their protein synthesis pathway has a relatively limited reserve capacity, making them more sensitive to fluctuations in eIF2α phosphorylation. In contrast, slow-twitch fibers have a lower basal translation rate and thus possess a greater compensatory capacity within their synthetic pathways.

#### Overactivation of protein degradation pathways accelerates selective muscle fiber loss

In sarcopenia, overactivation of the ubiquitin–proteasome system (UPS) and the autophagy-lysosome system is a key mechanism driving the degradation of muscle fiber-specific proteins and phenotypic transition, with a selective damaging effect on fast-twitch fibers [73, 81].

Within the ubiquitin–proteasome system, two genes—muscle RING-finger protein-1 (MuRF1) and muscle atrophy F-box protein (MAFbx), also known as Atrogin-1—are involved in skeletal muscle protein degradation [50]. MuRF1 specifically binds to and degrades the fast-twitch fiber MyHC IIa and IIx isoforms, whereas Atrogin-1 inhibits fast-twitch fiber formation by degrading myogenic regulatory factors [70]. The structural basis for this selectivity lies in the ability of the B-box domain of MuRF1 to recognize a unique linear motif at the carboxyl terminus of fast-twitch MyHC isoforms, whereas the slow-twitch MyHC type I isoform lacks this motif and is thereby protected from MuRF1-mediated ubiquitination and degradation [9]. Studies have shown that the mRNA expression levels of MuRF1 and Atrogin-1 in the gastrocnemius muscle of aged rats are elevated compared to young rats, and their expression levels are significantly negatively correlated with the cross-sectional area of type II muscle fibers [10]. In skeletal muscle biopsy samples from patients with sarcopenia, UPS activation results in an increased degradation rate of MyHC in type II muscle fibers compared to healthy controls, whereas no significant change is observed in the degradation rate of type I fibers, suggesting selective degradation of fast-twitch fibers by the UPS [27].

Dysfunction of the autophagy-lysosome system accelerates the transition process by disrupting muscle fiber homeostasis. Under normal physiological conditions, autophagy maintains muscle fiber function by clearing damaged organelles. However, age-related reduction in autophagic flux leads to the accumulation of metabolic waste and oxidative damage [47]. In the skeletal muscle of aged mice, the expression of key autophagy-related genes, such as Beclin-1 and microtubule-associated protein 1 light chain 3-II (LC3-II), is decreased, whereas the autophagy substrate p62 accumulates excessively. This results in mitochondrial dysfunction in fast-twitch fibers, thereby promoting their transition toward slow-twitch fibers [72]. Further studies have confirmed that FoxO3a-mediated downregulation of autophagy-related genes, including LC3 and BNIP3, exacerbates the deposition of aberrant proteins in fast-twitch fibers. Conversely, activation of the autophagy pathway significantly ameliorates the loss of fast-twitch fibers and increases MyHC IIa expression in aged mice [53].

#### Synergistic effects of mitochondrial dysfunction

Mitochondria, as the core hub of energy metabolism in muscle fibers, exhibit dysfunction that synergistically amplifies the imbalance in protein metabolism, with a particularly pronounced impact on type IIx fast-twitch fibers [22]. In sarcopenia, mitochondria display structural and functional abnormalities, including swelling, cristae disruption, and decreased membrane potential. Due to the lack of histone protection, mitochondrial DNA (mtDNA) is vulnerable to reactive oxygen species (ROS) attack, leading to the accumulation of mutations in genes encoding respiratory chain complexes, such as ND1 and COX1. This results in decreased activity of complexes I and IV and reduced ATP synthesis efficiency [22]. Type IIx fast-twitch fibers primarily rely on glycolysis for energy supply,therefore, mitochondrial dysfunction leads to insufficient energy availability during sustained contraction. In contrast, type I slow-twitch fibers depend on oxidative metabolism and can compensate through PGC-1α-mediated mitochondrial biogenesis to maintain basal energy supply. This difference in energy metabolism further drives the transition of type IIx fibers toward a slow-twitch phenotype. Additionally, oxidative stress resulting from mitochondrial dysfunction activates calcium-dependent proteases, which degrade myofibrillar proteins and exacerbate type IIx fiber atrophy.

Single-cell transcriptomic data have further validated these findings. In aging skeletal muscle, the expression of genes associated with motor neurons (MNs) innervating type IIx fibers is significantly downregulated, and the extent of MN loss is positively correlated with the degree of type IIx myonuclear loss [36]. In contrast, small-diameter MNs innervating type I fibers exhibit relatively stable mitochondrial function, with reduced ROS production and milder oxidative stress damage,accordingly, the downregulation of their associated genes is less pronounced [23]. Although MNs innervating type IIa fibers are also affected by oxidative stress and inflammation, the degree of damage is less severe than that in large-diameter MNs, and they retain some capacity for axonal sprouting and reinnervation.

### Inflammaging

Following intracellular anabolic-catabolic imbalance-induced damage to type IIx muscle fibers, inflammaging of the muscular microenvironment and extracellular matrix remodeling act synergistically to amplify muscle fiber damage signals and accelerate the transition of type IIx fibers [36, 44].

In sarcopenia, inflammaging manifests as a chronic low-grade inflammatory state, fundamentally characterized by an imbalance between pro-inflammatory and anti-inflammatory mechanisms [63]. Age-related inflammaging is characterized by elevated levels of pro-inflammatory cytokines, including TNF-α and IL-6 [32]. TNF-α activates the nuclear factor-κB (NF-κB) pathway, which upregulates the expression of MuRF1 and Atrogin-1, thereby accelerating the degradation of fast-twitch fibers [37]. Meanwhile, IL-6 suppresses the IGF-1/PI3K/Akt pathway, reducing mTOR activity and inhibiting protein synthesis in fast-twitch fibers [25]. Animal studies have shown that knockout of the IL-6 gene significantly ameliorates fast-twitch fiber loss and maintains MyHC IIa expression in aged mice, whereas administration of recombinant IL-6 accelerates the fast-to-slow fiber type transition [3]. Additionally, inflammatory factors exacerbate the imbalance between protein synthesis and degradation by inducing oxidative stress [30]. However, it should be noted that systemic IL-6 knockout may indirectly affect skeletal muscle through alterations in whole-body metabolism and immune status, and the specific cell-type-specific mechanisms remain unclear. Moreover, the translatability of this intervention strategy to humans remains to be evaluated.

Single-cell transcriptomic analysis has revealed that macrophages constitute the most abundant immune cell population in the skeletal muscle immune microenvironment of patients with sarcopenia, with four distinct subtypes identified. The functional imbalance of these macrophages is a key driver of chronic inflammation [58, 63]. Damage-associated molecular patterns (DAMPs) released by injured muscle fibers activate the Toll-like receptor 4 (TLR4) pathway, recruiting circulating monocytes and promoting their differentiation into M1 macrophages. These M1 macrophages subsequently recruit additional inflammatory cells via the CCL2-CCR2 axis and release pro-inflammatory cytokines, including TNF-α, IL-6, and IL-1β [19, 36]. TNF-α activates IKKβ kinase, leading to phosphorylation of the NF-κB p65 subunit. Upon nuclear translocation, p65 binds to κB sites in the MyHC IIx promoter, directly suppressing MyHC IIx gene transcription [36]. IL-6, via activation of the STAT3 pathway, promotes the expression of MuRF1 and MAFbx, thereby accelerating muscle protein degradation [44]. Following neutrophil infiltration, neutrophil extracellular traps (NETs) are released. NETs are composed of histone H3, neutrophil elastase (NE), and matrix metalloproteinase 9 (MMP-9). NE within NETs directly degrades sarcolemmal proteins, while MMP-9 disrupts NMJ structure, thereby exacerbating the denervation process [4]. Notably, clinical observations suggest potential sex differences in the rate and extent of muscle fiber type transition. Males may exhibit a more pronounced reduction in the cross-sectional area of type IIx fibers, whereas postmenopausal females may demonstrate a greater propensity for inflammaging [13].

## Exercise intervention strategies targeting muscle fiber type transition

### Molecular mechanisms and effects of targeted exercise modalities

The pathological progression of sarcopenia exhibits pronounced individual variability. Therefore, core intervention principles must accommodate both differences in physiological reserve across the aging spectrum and the specificity of molecular targets associated with the predominant phenotypic presentation. Based on the molecular targets involved in muscle fiber type transition, the strategic selection of exercise modalities—such as resistance training, aerobic exercise, or high-intensity interval training (HIIT)—can achieve specific repair of muscle fiber damage. Among these, resistance training represents the core modality for reversing type II fast-twitch fiber atrophy, whereas aerobic exercise and HIIT serve as key modalities for preserving the oxidative function of type I slow-twitch fibers [12, 13].

### Resistance training reverses type II fiber atrophy

Resistance training (RT) directly targets the core pathological mechanisms underlying type II fast-twitch fiber atrophy through mechanical stress stimulation of skeletal muscle, establishing it as the preferred exercise modality for reversing type II fiber loss in sarcopenia [21].

Mechanical stress induced by resistance training activates integrin α7β1 on the sarcolemma, triggering PI3K/Akt signaling. This upregulates the expression of the branched-chain amino acid transporter LAT1, facilitating leucine entry into myocytes and specifically activating the mTORC1 complex [13, 65]. The molecular basis for this selectivity lies in the fact that type II muscle fibers express significantly higher levels of the integrin β1 subunit than type I fibers, and their phosphatidic acid levels are more sensitive to mechanical tension, resulting in a more potent mTORC1 activation signal upon loading stimulation. In contrast, type I fibers, which express high levels of AMPK, exhibit a relatively attenuated anabolic response to mechanical stress. A clinical study has demonstrated that 12 weeks of progressive resistance training upregulates MyHC-IIx expression and increases the cross-sectional area of type II fibers in patients with sarcopenia [21]. One study has suggested that a resistance training regimen consisting of 60–80% of one-repetition maximum (1RM), performed 2–3 times per week with 2–3 sets per session, effectively activates mTORC1-mediated protein synthesis and suppresses MuRF1 expression. Furthermore, a cumulative training volume of approximately 1000 min or more is associated with a more pronounced increase in muscle fiber cross-sectional area [66]. However, it should be noted that this dose–response relationship may vary across elderly populations with different ages, sexes, and baseline physical conditions. Therefore, clinical prescription should be dynamically adjusted based on individual tolerance.

Resistance training induces Akt-mediated phosphorylation of FoxO1 and FoxO3, inhibiting their nuclear translocation and subsequent transcriptional activity. This downregulates the expression of the muscle-specific ubiquitin ligases MuRF1 and MAFbx, thereby blocking the ubiquitin–proteasome system-mediated degradation of structural proteins in type II fibers [13]. Concurrently, Akt suppresses the phosphorylation of Smad3 in the TGF-β/Smad3 pathway, reducing myofibroblast activation and extracellular matrix collagen deposition. This alleviates the physical barrier that fibrosis imposes on the regeneration of type II fibers [44]. Furthermore, resistance training promotes the polarization of M2 macrophages, reducing the mRNA levels of TNF-α and IL-1β in muscle tissue and mitigating the inflammatory microenvironment-induced damage to type II fibers [40]. The mechanical stress generated by resistance training also acts synergistically with mTORC1 activation. This synergy promotes the activation of quiescent satellite cells, downregulates the expression of inflammatory genes such as FOS and JUN, and reduces the premature entry of satellite cells into a primed state, thereby preventing pool depletion. Ultimately, this enhances the efficiency of satellite cell differentiation toward type II myoblasts [36, 63].

### Aerobic exercise and high-intensity interval training preserve type I fibers

Aerobic exercise and high-intensity interval training (HIIT) primarily target type I slow-twitch fibers, delaying the decline in oxidative function and preventing their aberrant transition. Concurrently, these modalities provide metabolic protection for type II fibers, rendering them suitable for sarcopenic patients presenting with endurance decline or combined endurance and muscle mass decline [12, 22].

Aerobic exercise increases the intracellular AMP/ATP ratio, which specifically activates AMPKα2. This, in turn, phosphorylates PGC-1α, promoting its interaction with NRF1 and ERRα to form a transcriptional complex. This complex upregulates the expression of genes involved in mitochondrial biogenesis and oxidative metabolism [22, 39]. In contrast, the key distinction of HIIT lies in its intermittent high-intensity load, which stimulates AMPK through both mechanical stress and energy stress during exercise, whereas during the recovery period following exercise, the sustained presence of amino acid supply and mechanical signals shifts the activation toward the mTORC1 pathway. This temporal uncoupling of AMPK and mTORC1 activation enables HIIT to promote mitochondrial biogenesis while simultaneously maintaining muscle protein synthesis. In contrast, aerobic exercise, which lacks mechanical stress stimuli of sufficient intensity, exhibits significantly lower levels of mTORC1 activation. This differential activation pattern of signaling pathways explains the potential additional advantage of HIIT over continuous aerobic exercise in preserving muscle mass. A longitudinal aging study has shown that 16 weeks of moderate-intensity brisk walking training enhances the activity of mitochondrial complex IV in the skeletal muscle of patients with sarcopenia, increases oxidative phosphorylation efficiency in type I fibers, and improves endurance performance [22].

HIIT achieves dual activation of the AMPK and mTORC1 pathways, concurrently optimizing oxidative function in type I fibers and preserving protein synthesis in type II fibers. Moreover, the total exercise duration is reduced by 50% compared to continuous aerobic exercise, making HIIT particularly suitable for elderly and very old patients with sarcopenia [12]. However, the anabolic stimulus of HIIT on type II fibers largely depends on the nutritional supply during the recovery period,inadequate protein intake may significantly attenuate its muscle-sparing effect. During the high-intensity intervals, AMPK activity is rapidly elevated, promoting mitochondrial biogenesis. Nuclear PGC-1α protein content increases significantly three hours post-exercise, and mitochondrial protein content and enzyme activity continue to rise for up to 24 h [61]. During the recovery periods, mTORC1 is activated, moderately stimulating muscle protein synthesis and thereby preventing the muscle mass loss associated with aerobic exercise alone. Clinical studies have demonstrated that HIIT significantly enhances the fatigue resistance of type I fibers in patients with sarcopenia while simultaneously suppressing the transition of type II fibers toward an excessive glycolytic phenotype, thereby achieving synergistic protection across different muscle fiber types [12].

In addition to exercise interventions, emerging strategies such as nutritional and pharmacological approaches offer potential directions for the prevention and treatment of sarcopenia. Regarding nutritional interventions, eicosapentaenoic acid (EPA) has been reported to improve muscle function by modulating muscle fiber type transition [78]. Animal studies suggest that long-term caloric restriction attenuates age-related atrophy in both slow-twitch and fast-twitch fibers of the rat soleus muscle [17, 48]. However, the feasibility and safety of caloric restriction in humans require rigorous evaluation. In terms of pharmacological interventions, animal model studies have shown that melatonin ameliorates age-related sarcopenia by inhibiting the fibrogenic conversion of satellite cells [24], and targeting the DP2 receptor attenuates muscle atrophy by inducing oxidative muscle fiber transition [51]. Nevertheless, the above pharmacological intervention strategies currently lack evidence from human clinical trials, and their efficacy and safety in patients with sarcopenia remain to be validated.

## Conclusion

Muscle fiber type transition is a core pathological hallmark of age-related sarcopenia, characterized by the shift from type II fast-twitch fibers to type I slow-twitch fibers, with the type IIx subtype being particularly affected. Available evidence suggests that disrupted neural input may serve as an initiating factor: selective loss of large-diameter α-motor neurons, degeneration of the neuromuscular junction, and dysregulation of neurotrophic support collectively contribute to the denervation of type IIx fibers and the initiation of the transition program. Regarding the selective vulnerability between fast- and slow-twitch fibers, large-diameter motor neurons are more susceptible to damage due to their long axons, high metabolic demands, and sensitivity to ROS. Type II fibers are the primary targets of degradation because MuRF1 selectively recognizes fast-twitch MyHC isoforms. Furthermore, the intermittent discharge pattern of type IIx fibers fails to provide sufficient survival signals. Collectively, these factors render fast-twitch fibers more severely affected.

Intracellular protein metabolic imbalance constitutes a key hub in this process, characterized by suppression of mTOR-mediated synthetic pathways, overactivation of the ubiquitin–proteasome and autophagy-lysosome systems, and superimposed mitochondrial dysfunction, collectively promoting fast-twitch fiber degradation while conferring a slow-twitch phenotype. Inflammaging and extracellular matrix remodeling form a vicious cycle, and the epigenetic network may participate in stabilizing the aberrant phenotype. Notably, the proportion of type IIa-IIx hybrid fibers in aged skeletal muscle is significantly increased, with a transition pattern lying between pure type IIx and pure type I fibers, potentially serving as a transitional reserve pool during the conversion process. However, systematic studies on the motor neuron matching of hybrid fibers, their vulnerability to denervation, and the impact of exercise interventions are currently lacking.

Existing clinical studies suggest that exercise interventions hold potential for ameliorating muscle fiber transition. Resistance training is associated with activation of the PI3K/Akt/mTOR pathway and inhibition of FoxO, correlating with the reversal of type II fiber atrophy observed clinically. Aerobic exercise and high-intensity interval training are associated with activation of the AMPK/PGC-1α pathway and optimization of mitochondrial function, correlating with a protective effect on the type I phenotype. The above dose–response relationships and optimal intervention protocols require validation by randomized controlled trials. It should be noted, however, that the majority of molecular mechanisms discussed in this review are primarily based on rodent models. Given the differences between rodents and humans in skeletal muscle fiber type composition, motor unit discharge patterns, and metabolic rate, direct extrapolation of findings from animal experiments to human sarcopenia should be made with caution. Future human functional studies are needed for validation.

## Statement

This work was supported by the Non-profit Central Research Institute Fund ofChinese Academy of Medical Sciences (Grant No. 2020-JKCS-022).

## Data Availability

No datasets were generated or analysed during the current study.
